# Treatment of Joint Diseases

**Published:** 1863-05

**Authors:** 


### TREATMENT OF JOINT DISEASES.

H. G. DAVIS, M. D., of New York, concludes an essay on Treatment of Joint Diseases with the following:

*First.*—That in all diseases exterior to the joint, when of sufficient gravity to render the same functionally immovable, and when continued for any length of time, the cartilages should be relieved from pressure by extension.

*Secondly.*—When the disease is within the capsular ligament, extension should be applied from the commencement, as the destruction of the cartilage will be in proportion to the activity of the inflammatory process.

*Thirdly.*—In immobility of the joints, from whetever cause, change of position of the articulating surfaces must be frequent, or extension applied. This extension should always be made by means of a cord, pulley, and weight, or by some *elastic* material, *the result of both being that a certain amount of extension is kept up, whatever may be the position of the limb.*

This is never fully accomplished by fixing a limb in a given position, as by the ordinary splint, with its so-called extension. When extension is made by an elastic material the muscular fibre becomes wearied, the nervous influence is expended, and the bones come down until the extending power is exerted entirely upon the unyielding tissues. There is, practically, a radical distinction between fixing a limb as by the old mode of extension, and that by which an unremitting draft is kept up upon the muscles, and yet the limb is not so fixed but that the muscles may contract and thereby exhaust their nervous influence, and ultimately rest like any muscle wearied from exercise.

Previous to my introducing it, I have never seen elastic extension recommended, except by one author, and he advised it simply for the purpose of preventing surgical apparatus in fractures from loosening, and not for the reason for which I use it, viz., for overcoming muscular contraction.

It will not be amiss to state what application of the principle of what I term "continued elastic extension," I have made, and with what results.

In ulcerations of the intervertebral cartilages and of the bodies of the vertebræ, I have devised apparatus that separates the diseased vertebræ from each other, and impoess the labor of sustaing the weight perpendicularly upon the lateral processes. Here it can rest until the disease stops, and the cavity is filled by bone. With this treatment restoration takes place with a good figure.

For morbus coxarius I have originated the treatment by appropriate splints, with which most of the members of the Academy are acquainted. The management of hip disease, based upon this principle, relieves entirely the suffering, puts the parts in the best condition for perfect restoration, and even if the disease is not checked, the limb is kept at full length and in a correct position.

Affections of the knee I treat in a similar manner and with the same success. I have devised an apparatus that will keep up extension upon this joint, and yet admit of flexion of the limb, the whole weighing but a few ounces.

The principle of the latter apparatus is also made applicable to the elbow and other joints.

